# Impact of Eliminating Cost-Sharing by Medicare Beneficiaries for Follow-Up Colonoscopy After a Positive Stool-based Colorectal Cancer Screening Test

**DOI:** 10.1158/2767-9764.CRC-23-0322

**Published:** 2023-10-17

**Authors:** A. Mark Fendrick, David Lieberman, Jing Voon Chen, Vahab Vahdat, A. Burak Ozbay, Paul J. Limburg

**Affiliations:** 1 Department of Internal Medicine and Department of Health Management and Policy, Division of General Medicine, University of Michigan, Ann Arbor, Michigan.; 2 Division of Gastroenterology and Hepatology, School of Medicine, Oregon Health and Science University, Portland, Oregon.; 3 Exact Sciences Corporation, Madison, Wisconsin.

## Abstract

Medicare coverage of a follow-up colonoscopy after a positive stool-based colorectal cancer screening test with no patient cost-sharing started January 2, 2023, which may favorably affect screening behavior. This analysis estimated the clinical and economic effects of increased colorectal cancer screening participation potentially resulting from this policy change in Medicare beneficiaries. The validated Colorectal Cancer and Adenoma Incidence & Mortality (CRC-AIM) model simulated three guideline-endorsed colorectal cancer screening strategies for average-risk individuals (colonoscopy every 10 years, annual fecal immunochemical test, triennial multitarget stool DNA) from ages 65–75 years. The base-case scenario assumed 0% coinsurance for initial screening and follow-up colonoscopy, real-world screening test use (colonoscopy = 45.3%, stool-based test = 24.4%, unscreened = 30.3%), and real-world follow-up colonoscopy rates. Comparative scenarios assumed an increase in the overall screening rate from 0% to 15% (5% increments) and an increase in the follow-up colonoscopy rate from 0% to 15% (5% increments). The base-case scenario resulted in 128 life-years gained (LYG)/1,000 individuals versus no screening and total screening and treatment costs of $7,938/person. The changes resulted in an increase of up to 26 LYG/1,000 individuals and a decrease in total screening and treatment costs by as much as $128/person. Follow-up colonoscopy at $0 coinsurance became cost-saving with any increase in either overall screening or follow-up colonoscopy. Policies that remove cost barriers to completing colorectal cancer screening may increase rates of screening participation, potentially improving economic and clinical outcomes.

**Significance:**

A follow-up colonoscopy after a positive stool-based colorectal cancer screening test is necessary to complete the full screening process. Policies that remove cost barriers to completing colorectal cancer screening may lead to increases in overall participation rates and use of follow-up colonoscopy, improving clinical and economic outcomes.

## Introduction

Colorectal cancer is estimated to claim approximately 53,000 lives in the United States in 2023 (1). Screening for colorectal cancer reduces the incidence and mortality of colorectal cancer (2, 3) and is suggested for all average-risk individuals ages 45–75 years (4–6). Patients have a variety of endorsed screening options to choose from including colonoscopy and stool-based tests [i.e., multitarget stool DNA (mt-sDNA) or fecal immunochemical test (FIT)] among others (4–6). If patients choose a stool-based screening option, a positive test result should be followed up with a colonoscopy to identify or exclude any colorectal cancer or precancerous neoplasia (4–6). Rates of follow-up colonoscopy completion demonstrate room for improvement, and failure to complete the follow-up colonoscopy negates the potential benefits of stool-based screening. A retrospective study of patients with a positive FIT test found that the risk of dying from colorectal cancer was doubled in those who did not have a follow-up colonoscopy after the positive test compared with those who did have a follow-up colonoscopy (7).

Despite the proven benefits of colorectal cancer screening, adherence to recommended screening ages and intervals is less than perfect and varies by screening test (8–10). There are many factors identified by patients for not undergoing initial colorectal cancer screening or follow-up colonoscopies in accordance with guideline recommendations, one of which is cost (11–13). Patient cost-sharing for initial colorectal cancer screening has been eliminated for those enrolled in commercial insurance plans and Medicare since 2011. Encouragingly, removal of patient cost-sharing was associated with increased screening adherence in some populations (14–17). However, out-of-pocket costs have remained for follow-up colonoscopies after a positive stool-based test. In a 2021 analysis of Medicare beneficiaries, 78% had out-of-pocket costs for a follow-up colonoscopy, and the costs increased when a polypectomy was performed (18). Recently, the Centers for Medicare and Medicaid Services (CMS) finalized their decision to cover follow-up colonoscopy after a positive stool-based colorectal cancer screening test with no cost-sharing for Medicare patients that went into effect on January 2, 2023 (https://www.cms.gov/newsroom/fact-sheets/calendar-year-cy-2023-medicare-physician-fee-schedule-final-rule). The clinical and economic impact of this new Medicare Physician Fee Schedule rule is unknown, although a recent study found that a policy that eliminated patient cost-sharing for follow-up colonoscopy after a positive stool-based test in Oregon significantly increased the overall uptake of colorectal cancer screening and shifted screening modalities from colonoscopy to noninvasive methods (19). The objective of this analysis was to estimate the clinical and economic effects from increased colorectal cancer screening that may stem from the recent CMS policy change eliminating patient cost-sharing for a follow-up colonoscopy after a positive stool test in a cohort of Medicare beneficiaries.

## Materials and Methods

### Microsimulation Model

The validated Colorectal Cancer and Adenoma Incidence & Mortality (CRC-AIM) microsimulation model was used for the analysis (20, 21). CRC-AIM predicts colorectal cancer outcomes based on colorectal cancer natural history and screening assumptions, and the outcomes are compared with an unscreened population or the base-case scenario. The natural history assumptions simulate the adenoma-carcinoma sequence in unscreened individuals and include age- and sex-specific risk of adenoma development, adenoma growth and chance of transitioning to preclinical and symptomatically detectable colorectal cancer, which triggers colorectal cancer survival based on sex, age, cancer location, and stage at detection. Details of the natural history assumptions have been described previously (20, 21). The screening inputs for the model include: type of screening test and associated performance characteristics (e.g., sensitivity and specificity), initial screening test adherence, follow-up colonoscopy adherence (when indicated), and population age.

An average-risk U.S. population of 2 million individuals was modeled that received screening from ages 65–75 years with colonoscopy every 10 years, mt-sDNA every 3 years, or annual FIT. The base-case scenario assumed 0% coinsurance for initial screening and follow-up colonoscopy after a positive mt-sDNA or FIT test.

### Screening Inputs

The base-case scenario weight-averaged the estimated outcomes by real-world screening test use determined from a U.S. longitudinal claims database study [colonoscopy = 45.3%, stool-based test = 24.4% (10.7% mt-sDNA, 13.7% FIT), unscreened = 30.3%] (22). Real-world rates for follow-up colonoscopy were assumed to be 71.5% after positive mt-sDNA (23) and 46.7% after positive FIT (23).

Outcomes from the base-case scenario were compared with scenarios that assumed potential modifications to screening behavior in response to the coinsurance elimination. These modifications were an increase in the base-case overall screening rate ranging from 0% to 15% in 5% absolute increments and an increase in the base-case follow-up colonoscopy rate ranging from 0% to 15% in 5% absolute increments. Outcomes were calculated separately for colonoscopy, mt-sDNA, and FIT and then aggregated to obtain total results.

Additional scenarios were modeled that also assumed a screening behavior change in base-case initial screening test use from colonoscopy to stool-based tests (5% or 10% absolute shift; i.e., shift from 45.3% to 40.3% or 35.3%, respectively, for colonoscopy and from 24.4% to 29.4% or 34.4%, respectively, for stool tests corresponding to initial screening). The potential 5% or 10% shift from colonoscopy to stool-based tests was distributed between mt-sDNA and FIT by their recently reported utilization patterns among stool-based test users (44% mt-sDNA, 56% FIT; ref. 22).

Performance characteristics for the screening tests in all scenarios were identical to those used by the U.S. Preventive Services Task Force 2021 decision analysis for colorectal cancer screening (Supplementary Table S1; ref. 24; https://www.uspreventiveservicestaskforce.org/uspstf/document/final-modeling-report/colorectal-cancer-screening).

### Cost and Utility Inputs

Cost inputs were estimated Medicare costs in 2022 U.S. dollars. Cost inputs for colorectal cancer screening, colonoscopy complications, and colorectal cancer–related medical care (initial, continuous, and terminal care for stages I–IV) and their source references are listed in Supplementary Table S2. Utility inputs and their source references are listed in Supplementary Table S3.

### Outcomes

Outcomes were life-years gained (LYG) per 1,000 individuals, total costs per person in U.S. dollars, and the incremental cost-effectiveness ratio (ICER). Total costs included screening costs, costs from colonoscopy complications, and colorectal cancer–related medical costs. Screening costs included stool-test and follow-up colonoscopy costs. The ICER threshold for cost-effectiveness was $100,000 per quality-adjusted life year.

### Data Availability

The data generated in this study are available within the article. CRC-AIM demonstrates the approach by which existing colorectal cancer models can be reproduced from publicly available information and provides a ready opportunity for interested researchers to leverage the model for future collaborative projects or further adaptation and testing. To promote transparency and credibility of this model, CRC-AIM's formulas and parameters are available on a public repository (https://github.com/CRCAIM/CRC-AIM-Public).

## Results

**FIGURE 1:**
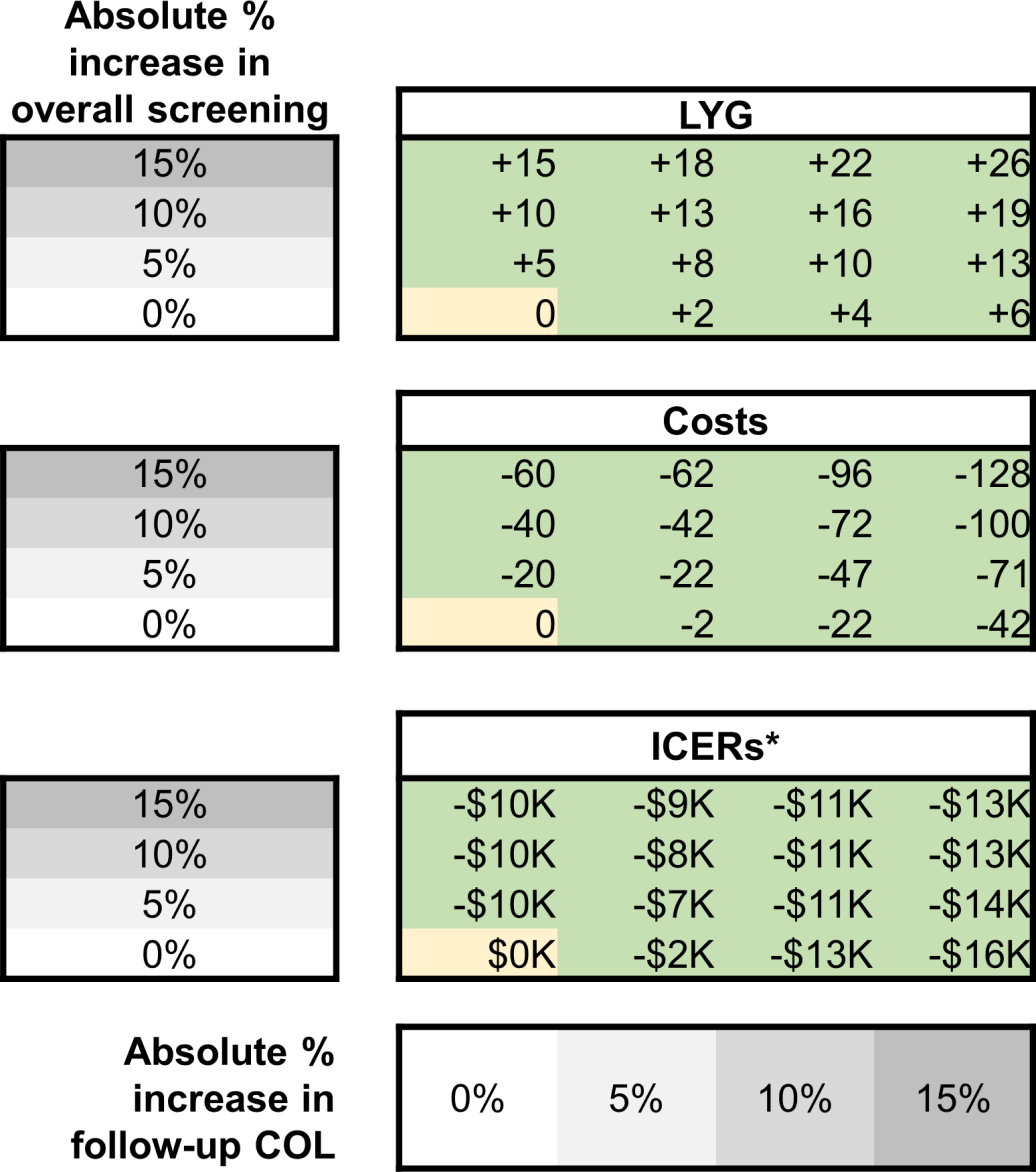
Incremental LYG, incremental total screening and treatment costs, and ICERs by scenario compared with the base-case scenario. The figure depicts the incremental LYG per 1,000 individuals compared with the base-case scenario (LYG = 128), the incremental total costs per person compared with the base-case scenario ($7,938), and ICERs when assuming increases in the overall screening rate by 0%–15% in 5% absolute increments and increases in the follow-up colonoscopy rate by 0%–15% in 5% absolute increments. Green indicates an increase in LYG or lower costs versus the base-case scenario. For the ICERs, green boxes indicate the scenario is cost-saving against the base-case scenario at a threshold of $100,000 per quality-adjusted life year. *Negative ICERs indicate the scenario is less expensive but more effective than (i.e., dominates) the base-case scenario. COL, colonoscopy; ICER, incremental cost effectiveness ratio; LYG, life-years gained.

The base-case scenario resulted in 128 LYG per 1,000 individuals compared with no colorectal cancer screening and a total combined colorectal cancer screening and treatment cost of $7,938 per person. Figure 1 portrays the impact on LYG and costs when assuming 0% cost-sharing changes the overall screening rate (0%, 5%, 10%, 15%) and changes the follow-up colonoscopy rate after a stool-based test (0%, 5%, 10%, 15%). The changes resulted in an increase of up to 26 LYG per 1,000 individuals and a decrease in total screening and treatment costs by as much as $128 per person. Follow-up colonoscopy at $0 coinsurance became cost-saving with any increase in either overall screening or follow-up colonoscopy (Fig. 1).

When assuming that $0 coinsurance also resulted in a 5% or 10% shift in initial screening test use from colonoscopy to stool-based tests, the screening behavior changes resulted in an increase of up to 21 LYG per 1,000 individuals and a decrease in total colorectal cancer screening and treatment costs by as much as $96 per person (Supplementary Fig. S1). When assuming a 10% shift to stool-based testing, $0 coinsurance for a follow-up colonoscopy became cost-effective when overall screening increased by at least 15% and follow-up colonoscopy increased by at least 10% (Supplementary Fig. S1).

## Discussion

The frequency and amount paid by patients undergoing a follow-up colonoscopy after a positive noninvasive initial colorectal cancer screening test was one factor in the 2023 implementation of a federal policy to eliminate cost sharing for Medicare beneficiaries receiving this critical part of the colorectal cancer screening process. This policy that removes cost barriers to completing colorectal cancer screening may lead to increases in overall participation rates and use of follow-up colonoscopy. Validation of the assumed increases in screening participation rates will need to be examined in future real-world studies.

Policies that eliminate patient cost-sharing for follow-up colonoscopies have been limited to a few states and have been in effect since 2016. The only published real-world study determined colorectal cancer screening rates in Oregon and Kentucky after state-level polices removed cost-sharing for follow-up colonoscopies and then compared the results with neighboring states without similar policies (19). In Oregon, removing cost-sharing significantly increased the rate undergoing any colorectal cancer screening by 1% and was associated with a significant shift from colonoscopy to a noninvasive screening test for initial screening (e.g., 10% shift from colonoscopy to a noninvasive test). There was no increase in the rate of follow-up colonoscopy after a positive noninvasive test in Oregon, and there were no significant changes in any of the evaluated screening behaviors in Kentucky. These observations are based on limited experience to date given that the state-based policy changes are relatively recent. Nonetheless, the screening patterns observed in Oregon warranted the use of the validated CRC-AIM model to estimate the clinical and economic impact of possible changes in screening behaviors by Medicare beneficiaries in response to elimination of cost barriers for follow-up colonoscopy. Results from this simulation suggest that the LYG and costs of screening and treatment are intimately related to changes in overall screening and follow-up colonoscopy rates. Increases in screening rates after the elimination of cost-sharing for a follow-up colonoscopy saves more lives, increases LYG, and reduces medical expenditures.

The 1% increase in initial screening observed in Oregon after cost-sharing for follow-up colonoscopies was removed is promising (19). Increases of up to 12% in colorectal cancer screening participation were demonstrated in some populations after policies were implemented in 2011 that eliminated the patient cost-sharing for initial screening (14–17). In particular, low-income individuals, male Medicare beneficiaries, Medicare beneficiaries who were below the poverty income, and individuals identifying as Hispanic, had significant increases in colorectal cancer screening (14–17). A review of studies that investigated the impact of eliminating cost-sharing in the United States for breast, colorectal, and cervical cancer screening strategies found that many of the studies demonstrated an increase in screening rates, particularly in specific populations such as low-income individuals or Medicare beneficiaries (25).

While more real-world data are needed, previous modeling analyses have estimated the effect on clinical outcomes when assuming that removing follow-up colonoscopy coinsurance increases screening rates in simulated Medicare populations. One model assumed that an increase in initial FIT screening rate from 60% to 65%, along with an increase in the follow-up colonoscopy rate of 80% to 85%, decreased estimated colorectal cancer deaths by 6% with an ICER of $3,747 (26). In the same model, an increase in the FIT screening rate of only 0.3% was cost-effective at a willingness to pay threshold of $100,000 (26). A CRC-AIM analysis demonstrated that increases in screening rates as little as 1% to initial screening or follow-up colonoscopy in response to removing follow-up colonoscopy coinsurance improved clinical outcomes and were cost-effective (27).

This analysis emphasizes the value of modeling for colorectal cancer screening as a range of potential behavioral screening shifts, which allowed for exploration of the interactions among the most impactful, and in some cases uncertain, variables that influence clinical and economic outcomes. Policies that remove cost barriers to completing colorectal cancer screening can increase overall participation rates for both initial and follow-up testing. These changes may improve both economic and clinical outcomes. Thus, policies and efforts to increase both total screening participation and follow-up colonoscopy rates are paramount to improving public health.

## Supplementary Material

Supplementary Table 1 Table S1. Performance characteristic assumptions for the screening tests.

Supplementary Table 2 Table S2. Model cost inputs and references. All costs were inflated to April 2022 US dollars.

Supplementary Table 3 Table S3. Model utility inputs and references.

Supplementary Figure 1 Figure S1. Incremental LYG, incremental total screening and treatment costs, and ICERs by additional scenarios compared with the base-case scenario.
