# Supplementary material for: Impact of Eliminating Cost-Sharing by Medicare Beneficiaries for Follow-Up Colonoscopy After a Positive Stool-based Colorectal Cancer Screening Test
Source: Cancer Res Commun. 2023 Oct 17;3(10):2113–7. doi: 10.1158/2767-9764.CRC-23-0322 (PMC10581033; doi:10.1158/2767-9764.CRC-23-0322)
Supplement: Supplementary Table 1 — Table S1. Performance characteristic assumptions for the screening tests. [file crc-23-0322-s01.docx]

**Supplemental Material**

**Table S1.** Performance characteristic assumptions for the screening tests.^1, 2^

| **Screening test characteristic** | **Colonoscopy (within reach, per lesion)**^a^ | **FIT (per person)** | **mt-sDNA (per person)** |
| --- | --- | --- | --- |
| Sensitivity for CRC | 0.95 | 0.74 | 0.94 |
| Sensitivity for adenomas ≥10 mm | 0.95 | 0.22 | 0.42 |
| Sensitivity for adenomas 6-9 mm | 0.85 | 0.07^c^ | 0.15^c^ |
| Sensitivity for adenomas 1-5 mm | 0.75 |  |  |
| Specificity | 0.86^b^ | 0.97 | 0.91 |
| Reach, % | 95 to end of cecum, remainder between rectum and cecum | Whole colorectum | Whole colorectum |
| Risk of complications (serious GI, other GI, and CV complications) | Age-specific risks | 0 | 0 |

CRC, colorectal cancer; CV, cardiovascular; FIT, fecal immunochemical test with a cutoff for positivity of 20 μg of hemoglobin per g of feces; GI, gastrointestinal; mt-sDNA, multitarget stool DNA test.

^a^The same test characteristics were assumed for screening, diagnostic follow-up, and surveillance colonoscopies.

^b^The lack of specificity with endoscopy reflects the detection of non-adenomatous polyps, which leads to unnecessary polypectomy, which is associated with an increased risk of complications.

^c^Sensitivity for persons with nonadvanced adenomas. The same sensitivity was assumed for adenomas between 1 to 5 mm and 6 to 9 mm.
