# Supplementary material for: Impact of Eliminating Cost-Sharing by Medicare Beneficiaries for Follow-Up Colonoscopy After a Positive Stool-based Colorectal Cancer Screening Test
Source: Cancer Res Commun. 2023 Oct 17;3(10):2113–7. doi: 10.1158/2767-9764.CRC-23-0322 (PMC10581033; doi:10.1158/2767-9764.CRC-23-0322)
Supplement: Supplementary Table 2 — Table S2. Model cost inputs and references. All costs were inflated to April 2022 US dollars. [file crc-23-0322-s02.docx]

**Table S2.** Model cost inputs and references. All costs were inflated to April 2022 US dollars.

| **Input parameter** | **Estimated Medicare Cost, $** | **Source Reference** |
| --- | --- | --- |
| CRC screening |  |  |
| Colonoscopy, screening | $1,540.17 | Pyenson et al, 2014^3^ |
| Colonoscopy, diagnostic follow-up | $1,540.17 | Pyenson et al, 2014^3^ |
| Colonoscopy, symptom | $1,540.17 | Pyenson et al, 2014^3^ |
| Colonoscopy, surveillance | $1,540.17 | Pyenson et al, 2014^3^ |
| mt-sDNA | $508.87 | 2022 Centers for CMS Clinical Laboratory fee schedule |
| FIT | $15.92 | 2022 Centers for CMS Clinical Laboratory fee schedule |
| Colonoscopy complications |  | Hathway et al, 2020^4^ |
| Gastrointestinal | $9,091.39 |  |
| Serious gastrointestinal | $25,918.04 |  |
| Cardiovascular | $11,655.72 |  |
| CRC medical care |  | Mariotto et al, 2020^5^ |
| Stage I |  |  |
| Initial care | $47,325.70 |  |
| Continuous care | $4,857.25 |  |
| Terminal care, CRC death | $95,503.77 |  |
| Terminal care, non-CRC death | $25,076.26 |  |
| Stage II |  |  |
| Initial care | $67,115.98 |  |
| Continuous care | $5,663.82 |  |
| Terminal care, CRC death | $107,660.88 |  |
| Terminal care, non-CRC death | $26,984.55 |  |
| Stage III |  |  |
| Initial care | $97,499.84 |  |
| Continuous care | $8,774.34 |  |
| Terminal care, CRC death | $112,716.58 |  |
| Terminal care, non-CRC death | $36,900.05 |  |
| Stage IV |  |  |
| Initial care | $144,889.15 |  |
| Continuous care | $41,539.75 |  |
| Terminal care, CRC death | $141,732.83 |  |
| Terminal care, non-CRC death | $88,763.67 |  |

CRC, colorectal cancer; FIT, fecal immunochemical test; mt-sDNA, multitarget stool DNA.
