# Supplementary material for: Impact of Eliminating Cost-Sharing by Medicare Beneficiaries for Follow-Up Colonoscopy After a Positive Stool-based Colorectal Cancer Screening Test
Source: Cancer Res Commun. 2023 Oct 17;3(10):2113–7. doi: 10.1158/2767-9764.CRC-23-0322 (PMC10581033; doi:10.1158/2767-9764.CRC-23-0322)
Supplement: Supplementary Table 3 — Table S3. Model utility inputs and references. [file crc-23-0322-s03.docx]

**Table S3.** Model utility inputs and references.

| **Input parameter** | **Utility Value** | **Source Reference** |
| --- | --- | --- |
| General health utility | EQ-5D population norms | Szende et al, 2014^6^ |
| CRC Screening |  | Goede et al, 2017^7^ |
| Colonoscopy, screening | -0.0055 |  |
| Colonoscopy, diagnostic follow-up | -0.0055 |  |
| Colonoscopy, symptom | -0.0055 |  |
| Colonoscopy, surveillance | -0.0055 |  |
| Colonoscopy complications |  |  |
| Gastrointestinal | -0.0384 |  |
| Serious gastrointestinal | -0.0384 |  |
| Cardiovascular | -0.0384 |  |
| CRC |  | Goede et al, 2017^7^ and Djalalov et al, 2014^8^ |
| Stage I-III initial care | -0.15 |  |
| Stage I-III continuous care | -0.1 |  |
| Stage I-III terminal care, CRC death | -0.29 |  |
| Stage I-III terminal care, non-CRC death | -0.1 |  |
| Stage IV initial care | -0.34 |  |
| Stage IV continuous care | -0.29 |  |
| Stage IV terminal care, CRC death | -0.29 |  |
| Stage IV terminal care, non-CRC death | -0.29 |  |

CRC, colorectal cancer.
