# Supplementary material for: Impact of Eliminating Cost-Sharing by Medicare Beneficiaries for Follow-Up Colonoscopy After a Positive Stool-based Colorectal Cancer Screening Test
Source: Cancer Res Commun. 2023 Oct 17;3(10):2113–7. doi: 10.1158/2767-9764.CRC-23-0322 (PMC10581033; doi:10.1158/2767-9764.CRC-23-0322)
Supplement: Supplementary Figure 1 — Figure S1. Incremental LYG, incremental total screening and treatment costs, and ICERs by additional scenarios compared with the base-case scenario. [file crc-23-0322-s04.docx]

**Supplemental Material**

**Supplemental Figure S1.** **Incremental LYG, incremental total screening and treatment costs, and ICERs by additional scenarios compared with the base-case scenario.** The figure depicts the incremental LYG per 1000 individuals compared with the base-case scenario (LYG=128), the incremental total costs per person compared with the base-case scenario ($7938), and ICERs when assuming increases in the overall screening rate by 0-15% in 5% absolute increments, increases in the follow-up colonoscopy rate by 0-15% in 5% absolute increments, and a shift in initial screening from colonoscopy to stool-based tests by 0-10% in 5% absolute increments. Green indicates an increase in LYG or lower costs versus the base-case scenario; red indicates a decrease in LYG or higher costs versus the base-case scenario. For the ICERs, green boxes indicate the scenario is cost-saving against the base-case scenario, yellow boxes indicate the scenario is cost-effective against the base-case scenario, gray boxes indicate the scenario is not cost-effective against the base-case scenario, and black boxes indicate the scenario is dominated by the base-case scenario at a threshold of $100,000 per quality-adjusted life year. *Negative ICERs indicate the scenario is less expensive but more effective than (i.e., dominates) the base-case scenario. COL, colonoscopy; ICER, incremental cost effectiveness ratio; LYG, life-years gained.

**References**

1. Knudsen AB, Rutter CM, Peterse EFP, Lietz AP, Seguin CL, Meester RG, et al. Colorectal cancer screening: An updated decision analysis for the U.S. Preventive Services Task Force. [Internet]. Rockville, MD: Agency for Healthcare Research and Quality; 2021 [cited 2023 January 30]. Available from: <https://www.uspreventiveservicestaskforce.org/uspstf/document/final-modeling-report/colorectal-cancer-screening>.

2. Knudsen AB, Rutter CM, Peterse EFP, Lietz AP, Seguin CL, Meester RGS, et al. Colorectal Cancer Screening: An Updated Modeling Study for the US Preventive Services Task Force. JAMA. 2021;325:1998-2011.

3. Pyenson B, Scammell C, Broulette J. Costs and repeat rates associated with colonoscopy observed in medical claims for commercial and Medicare populations. BMC Health Serv Res. 2014;14:92.

4. Hathway JM, Miller-Wilson LA, Jensen IS, Ozbay B, Regan C, Jena AB, et al. Projecting total costs and health consequences of increasing mt-sDNA utilization for colorectal cancer screening from the payer and integrated delivery network perspectives. J Med Econ. 2020;23:581-92.

5. Mariotto AB, Warren JL, Zeruto C, Coughlan D, Barrett MJ, Zhao L, et al. Cancer-Attributable Medical Costs for Colorectal Cancer Patients by Phases of Care: What Is the Effect of a Prior Cancer History? J Natl Cancer Inst Monogr. 2020;2020:22-30.

6. Szende A, Janssen B, Cabases J.Self-Reported Population Health: An International Perspective based on EQ-5D. Self-Reported Population Health: An International Perspective based on EQ-5D 2014. Szende A, Janssen B, Cabases J, editors. Dordrecht (NL)10.1007/978-94-007-7596-1_5

7. Goede SL, Rabeneck L, van Ballegooijen M, Zauber AG, Paszat LF, Hoch JS, et al. Harms, benefits and costs of fecal immunochemical testing versus guaiac fecal occult blood testing for colorectal cancer screening. PLoS One. 2017;12:e0172864.

8. Djalalov S, Rabeneck L, Tomlinson G, Bremner KE, Hilsden R, Hoch JS. A Review and Meta-analysis of Colorectal Cancer Utilities. Med Decis Making. 2014;34:809-18.
